# The effects of music & auditory beat stimulation on anxiety: A randomized clinical trial

**DOI:** 10.1371/journal.pone.0259312

**Published:** 2022-03-09

**Authors:** Adiel Mallik, Frank A. Russo

**Affiliations:** Department of Psychology, Ryerson University, Toronto, Ontario, Canada; Universiti Tunku Abdul Rahman, MALAYSIA

## Abstract

**Background and objectives:**

Music and auditory beat stimulation (ABS) in the theta frequency range (4–7 Hz) are sound-based anxiety treatments that have been independently investigated in prior studies. Here, the anxiety-reducing potential of calm music combined with theta ABS was examined in a large sample of participants.

**Methods:**

An open-label randomized controlled trial was conducted with participants taking anxiolytics (n = 163). Participants were randomly assigned using the Qualtrics randomizer algorithm, to a single session of sound-based treatment in one of four parallel arms: combined (music & ABS; n = 39), music-alone (n = 36), ABS-alone (n = 41), or pink noise (control; n = 47). Pre- and post-intervention somatic and cognitive state anxiety measures were collected along with trait anxiety, personality measures and musical preferences. The study was completed online using a custom application.

**Results:**

Based on trait anxiety scores participants were separated into moderate and high trait anxiety sub-groups. Among participants with moderate trait anxiety, we observed reductions in somatic anxiety that were greater in combined and music-alone conditions than in the pink noise condition; and reductions in cognitive state anxiety that were greater in the combined condition than in the music-alone, ABS-alone, and pink noise conditions. While we also observed reductions in somatic and cognitive state anxiety in participants with high trait anxiety, the conditions were not well differentiated.

**Conclusions:**

Sound-based treatments are effective in reducing somatic and cognitive state anxiety. For participants with moderate trait anxiety, combined conditions were most efficacious.

## Introduction

Anxiety has been steadily increasing, particularly in the adolescent and young adult populations in the past 24 years [1]. The economic cost of anxiety in the 1990’s in the United States was estimated to range from $42.3 billion to $46.6 billion [2,3]. COVID-19 pandemic lockdowns have further increased the prevalence of anxiety with U.S. adults being three times more likely to screen positive for anxiety disorders in April/May 2020 compared to 2019 [4]. In many cases the origins of anxiety can be traced to early stressful life events (ELS) that alter the function of the hypothalamic-pituitary-adrenal (HPA) axis [5]. ELS has the effect of negatively influencing an individual’s development affecting all spheres of an individual’s life: emotional, cognitive, behavioural, social and physical [6]. In these terms, it is likely that the COVID19 pandemic could be an ELS for many people during this time. The bulk of the evidence indicates that ELS often lead to permanent changes in the HPA axis and may develop into anxiety in adulthood [5]. Specifically, increased activity of the HPA axis is associated with hypercortisolemia and reduced inhibitory feedback [5]. Indeed, higher cortisol levels are present in people suffering from anxiety disorders [7,8]. This suggests that treatments that target cortisol and other components of the HPA axis may be potentially effective in treating anxiety.

Many anxiety treatments exist, including anti-anxiety medications (selective serotonin re-uptake inhibitors, serotonin-norepinephrine reuptake inhibitors, benzodiazepines) [9–12], cognitive strategies, behavioural approaches (cognitive behavioural therapy, exposure, relaxation), mindfulness and acceptance-based approaches [13–15]. However, response rates to anti-anxiety medication can be poor, many patients also can have negative side effects such as sexual dysfunction and it is difficult to predict reliably which patients will respond well and which will have a limited treatment response [10]. In more extreme cases, benzodiazepines are prescribed to treat anxiety, are taken on an as-needed basis, are overused and are physically and psychologically addictive, particularly if used over extended periods of time [12,16]. Between 1996 and 2013, there was an average annual increase in benzodiazepine prescription of 2.5% in the United States [11], which represents an increase in the quantity of benzodiazepines consumed from 1.1 to 3.6 kilograms per 100,000 adults. The overdose rate increased concurrently from 0.58 to 3.07 per 100,000 adults [11].

Although cognitive behavioural therapy has proven to be effective in treating anxiety [14,15], limited accessibility to treatment remains a challenge [13]. Additionally, the active anxiety treating component of cognitive behavioural therapy requires patients to face their fears without use of emotion modulation strategies [13]. Many patients are unwilling to tolerate the extreme discomfort this may cause and make the commitment to the therapeutic process [13].

Many anxiety sufferers do not respond to these standard treatment approaches, and many others face barriers to treatment such as lack of access to psychologists and other mental health professionals [13,17]. Therefore, it is extremely important to identify other approaches that may be useful as an alternative or supplement to mainline treatments. Specifically, there is a need for a cost effective, easy to use, easily deployable at scale, efficacious anxiety treatment free from the serious side effects present in anti-anxiety medications such as SSRIs, SNRIs, and benzodiazepines. Many people already use music to manage their mental health and sound-based anxiety treatments involving music show promise in fulfilling this need [18].

Music listening can reduce anxiety [19–23] and some evidence suggests that it may do so more effectively than anti-anxiety drugs such as midazolam [24]. This may be due to the neurochemical effects of music which include increased levels of endogenous opioids and dopamine [25–27]. There is also strong evidence to suggest that this also may be due to music’s ability to reduce cortisol in a natural setting (field study) [28], as well as preventing cortisol increases and in some cases reducing cortisol in stressful situations [29–31]. The bulk of the evidence suggests that music can be very effective in reducing self-reported anxiety in non-clinical samples according to a recent meta-analysis [32]. However, the same meta-analysis found a non-significant decrease in psychophysiological signals related to anxiety. The authors of the meta-analysis suggested that the discrepancy between self-reports and psychophysiology may have been due to the heterogeneity of the studies considered and an overall lack of rigorous methodological standards which may have biased the results [32]. One randomized control trial involving children with anxiety disorders found that Multi-Modal Music Therapy (MMT), which is combination of music therapy and cognitive behavioural therapy was more effective than normal treatment (a mixture of behavioural interventions, psychodynamic psychotherapy, nonspecific group therapy) according to the remission rates after treatment which persisted for four months [33].

The mood regulating properties of music may be enhanced if the mood of the music is matched to an individual’s initial emotional state before being changed to the target state [34]. This approach to sequencing music selections follows the iso principle, first proposed as a system of mood regulation supported by music [35]. The music recommendation system used in this research is supplied by LUCID Inc. (https://www.thelucidproject.ca) and employs the iso principle along with affective classification and reinforcement learning to cultivate affect-driven personalized music sequences [36]. The sequences are instrumental music integrated with nature sounds and were composed by LUCID’s music director/composer. Prior to the music treatment the participant inputs their current mood using the arousal and valence dimensions of the Russell Circumplex Model [37]. Based on this input as well as the target emotional state of reduced anxiety/increased feelings of calm, the machine learning algorithm within the application predicts the optimal sequence of tracks to produce mood induction in the listener from their current emotional state to the target state. This machine learning algorithm uses reinforcement learning techniques and is trained on real-world data correlating the quantitative features of musical excerpts and sequences alongside the emotional responses induced by them in listeners. From a conceptual point of view, this algorithm is based on the iso principle, a methodology used in music therapy to achieve mood induction that involves matching musical stimuli to a patient’s current mood and gradually changing the music in the direction of their desired mood state [34]. The iso principle has been indicated in prior research to be more effective than other musical sequences at reducing tension [38].

ABS is a non-invasive neuromodulatory technique which uses sound waves to produce combination tones, binaural beats, or monoaural beats in the alpha (8–13 Hz), beta (14–30 Hz), theta (4–8 Hz), gamma (30–50 Hz) or delta (1–4 Hz) frequency ranges with the intention of producing a neural frequency following response [39–41]. Combination tone signals can be generated by superposing two sine waves of neighboring frequencies. Monaural beat refers to the presentation of amplitude modulated beat signals to a single ear, or to both ears simultaneously. When the individual sine waves are presented dichotically, a binaural beat percept is produced [42]. For example, a two-tone exposure of 400 and 405 Hz presented to each ear separately will be experienced as a modulated wave of 5 Hz by the listener [43]. Theta and delta ABS may reduce anxiety and increase self-reported relaxation [44–47].

In this study, 163 participants taking anxiolytics were recruited using Prolific, an online participant pool platform. Participants were randomly assigned to one of the following four treatments using the Qualtrics randomizer algorithm: 1) Combined (music & ABS), 2) Music-alone, 3) ABS-alone, and 4) Pink noise (control). Music preferences, trait anxiety and personality traits have been confounding factors in previous studies involving music, ABS, and mental health [48–51]. Therefore, before their treatment session, participants completed the Short Test of Music Preferences (STOMP), the short form of the Eysenck Personality Questionnaire (EPQR) and the trait version of the State Trait Inventory for Cognitive and Somatic Anxiety (STICSA). The participants completed the following measures pre and post treatment: the state version of the STICSA, Positive and Negative Affect Scale (PANAS).

Our hypotheses were that the combined, music alone and ABS alone conditions would experience a greater reduction in somatic and cognitive state anxiety compared to the pink noise control condition. These hypotheses were pre-registered using the Open Science Framework (Registration DOI: https://doi.org/10.17605/OSF.IO/VHCA5) and were based upon previous studies showing that ABS and music listening are capable of reducing anxiety [19–24,44–47,52–54]. We had no specific predictions for moderate and high trait anxiety participants, but our preregistration noted our intention to recruit from both of these populations. Trait anxiety is a confounding factor in studies involving music [50,51]. Therefore, we separated the participants into moderate and high trait anxiety conditions according to prior criteria to determine whether moderate and high trait anxiety participants differ in their responses to the treatments [55]. Trait anxiety was measured by the State Trait Inventory for Cognitive and Somatic Anxiety (STICSA), this is not a diagnostic tool but is a quantitative assessment of perceived anxiety level. This experimental protocol received approval from the Ryerson Research Ethics Board (REB 2020–068) and was conducted in accordance with the ethical principles stated in the Declaration of Helsinki [56]. All participants gave their informed consent prior to their inclusion in the study.

## Methods

### Design

An open-label randomized controlled trial was conducted with participants taking anxiolytics (n = 163). Participants were randomly assigned using the Qualtrics randomizer algorithm, to a single session of sound-based treatment in one of four parallel arms: combined (music & ABS; n = 39), music-alone (n = 36), ABS-alone (n = 41), or pink noise (control; n = 47). The Qualtrics randomizer algorithm was instructed to evenly distribute participants amongst the four parallel arms. This clinical trial is retrospectively registered at clinicaltrials.gov. This study was initially registered at the Open Science Framework (Registration DOI: https://doi.org/10.17605/OSF.IO/VHCA5) prior to participant recruitment and commencement of the study but has been retrospectively registered at clinicaltrials.gov to adhere to PLOS One publication standards.

### Sample size

Sample size was determined based on an *a priori* power analysis based on two previous studies that examined how anxiety levels were affected by music and binaural beats [51,57] indicated that to achieve a power of 0.80 at a significance of p = 0.05, expected effect size (Cohen’s d) of 0.84, we would need a minimum sample size of 68 participants each from moderate and high trait anxiety populations. We stopped participant recruitment once we reached the sample minimum required by our *a priori* power analysis.

### Randomization and blinding

This study was conducted using Qualtrics online survey software (www.qualtrics.com). This is an open-label clinical trial, and investigators were not blinded to the treatment the participants ended up receiving. Participants were randomly assigned using the Randomizer algorithm in Qualtrics online survey software, to a single session of sound-based treatment in one of four parallel arms: combined (music & ABS; n = 39), music-alone (n = 36), ABS-alone (n = 41), or pink noise (control; n = 47). The Qualtrics randomizer algorithm was instructed to evenly distribute participants amongst the four parallel arms. Any variations in n in terms of treatment assignment was due to technical issues that led some participants to download the application multiple times even though they only completed the study once. This altered the counts for each treatment on the randomizer which affected treatment assignment. The investigators did not have access to any randomization sequence that may have been used by the Qualtrics randomization algorithm, ensuring allocation concealment.

### Participants

**Fig 1:**
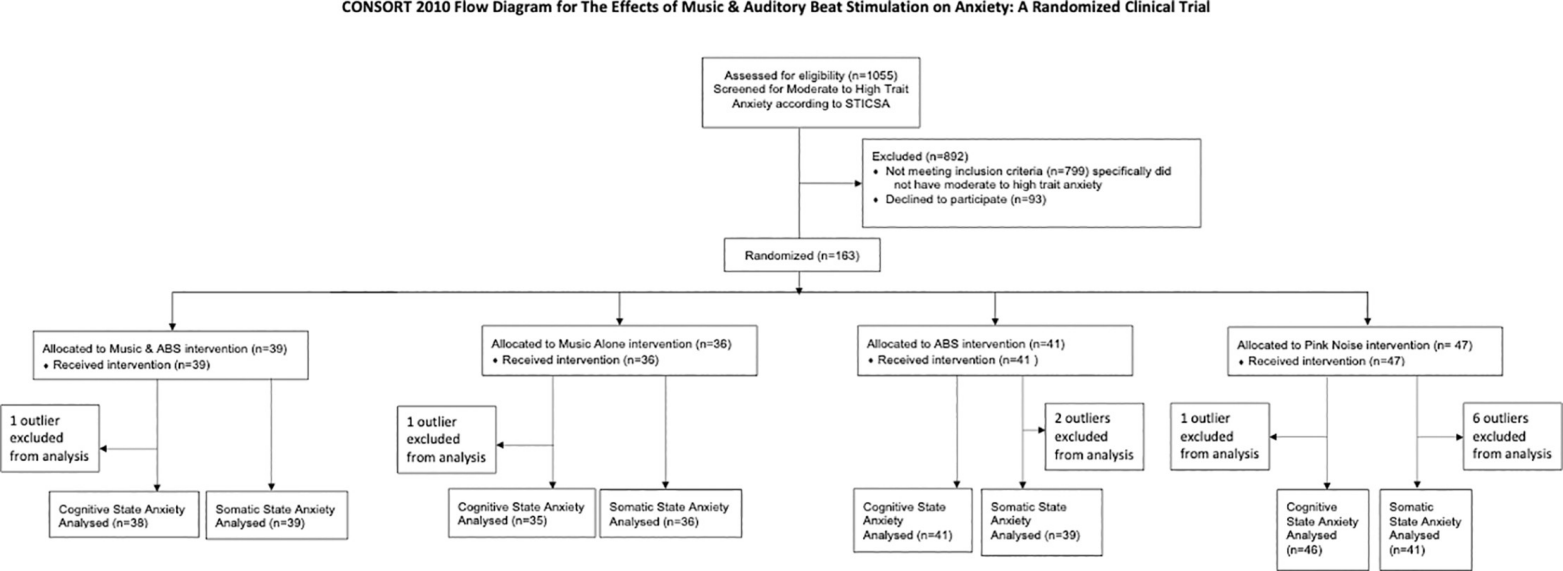
CONSORT participant flow diagram.

Participants (n = 1055) that were taking anxiolytics were assessed for trait anxiety using the online participant pool platform Prolific (www.prolific.co) (Fig 1). From this initial pool, those with moderate to high trait anxiety (n = 163, see Table 1 for demographic information of participants) were recruited using the online participant pool platform Prolific (www.prolific.co). Participant recruitment and study completion occurred from July 11^th^, 2020, to February 5^th^, 2021.

**Table 1:** Demographic information for moderate and high trait anxiety participants.

Moderate Trait Anxiety Participants		
Treatment	Gender Distribution	Mean Age (years)
Music & ABS (Combined)	8 males, 14 females	29.45
Music	3 males, 18 females	28.19
ABS	7 males, 16 females	26.35
Pink Noise	10 males, 17 females	31.33
High Trait Anxiety Participants		
Treatment	Gender Distribution	Mean Age (years)
Music & ABS (Combined)	3 males, 14 females	26
Music	4 males, 11 females	30.13
ABS	3 males, 15 females	25.59
Pink Noise	5 males, 15 females	28.3

This experimental protocol received approval from the Ryerson Research Ethics Board (REB 2020–068) and was conducted in accordance with the ethical principles stated in the Declaration of Helsinki [56]. All participants were over the age of 18 and gave their written informed consent prior to their inclusion in the study. Specifically, inclusion criteria were that participants had to be taking anxiolytics, have self-identified normal hearing, no known cardiac issues, no known epilepsy/seizures and had to have an iOS device (iPhone or iPad) to download and install the “LUCID Research Application” needed to participate in the study. From this participant population, moderate trait anxiety participants (n = 93) were classified as having a STICSA trait somatic score between 16.9 and 22.4, and a STICSA trait cognitive score between 17.1 and 26.6 [55]. High trait anxiety participants (n = 70) were classified as having a STICSA trait somatic score above 22.4 and a STICSA trait cognitive score above 26.6 [55].

### Self-report measures

The State Trait Inventory for Cognitive and Somatic Anxiety (STICSA) assessed somatic and cognitive trait and state anxiety in all the participants. The STICSA has good reliability and validity as a measure of state and trait cognitive and somatic anxiety [58,59].

The Positive and Negative Affect Scale (PANAS) was used to assess the mood of the participants before and after their randomly assigned treatment. The PANAS has good reliability and validity and has been widely used in many studies to assess mood [60,61].

The Short Test of Music Preferences (STOMP) assessed the musical preferences of the participants. The STOMP has good reliability and has been validated as a good measure of musical preferences [62].

The short form of the Eysenck Personality Questionnaire (EPQR) assessed the personality traits of participants, specifically their introversion, extraversion and neuroticism and has good reliability and validity [63].

### Treatment conditions

The music sequences in the music conditions were curated by the affective music recommendation system described in the introduction and deployed by the LUCID Research Application. This application is identical to the commercially available “LUCID Vibe Application” but has been specifically configured to allow for randomization of treatment conditions. All participants received 24 minutes of treatment and followed the same procedure independent of the experimental condition they were assigned to. The participants were randomly assigned to one of four treatment conditions: combined (music with ABS); music-alone, ABS-alone, or pink noise. Pink noise is noise that follows a 1/f energy distribution in the frequency domain [64]. Many prior studies involving auditory stimuli have used pink noise as a control stimulus [65–67]. Pink noise is not much different from a silent condition [68]. A potential issue in using a silent condition as a control is the potential for placebo effects since all other conditions have an auditory component. In addition, the advantage to pink noise as control condition compared to the silent condition is that helps control for the placebo effect. Pink noise was administered in the same manner as all the other stimuli, with the experimenter having control over this variable.

According to a prior meta-analysis study, the optimal treatment time for anxiety reduction and cognitive improvements for auditory beat stimulation and other music based treatments was 20–30 minutes [49]. The specific time of 24 minutes is due to a technical limitation involving the LUCID iso principle-based music sequencing machine learning algorithm.

### Procedure

This study was conducted online, and participants completed the study at their home. After consenting to the study, participants downloaded the LUCID Research Application on their iOS device. Participants then completed the pre-treatment survey consisting of the STICSA trait, the Eysenck Personality Questionnaire (EPQR), the Short Test of Music Preferences (STOMP), the Positive and Negative Affect Scale (PANAS), and the STICSA state. Participants then listened to their assigned treatment for 24 minutes. Participants were asked to use headphones and close their eyes while listening to the audio treatment. Participants then completed the post-treatment survey consisting of the STICSA state and the PANAS.

### Statistical methods

Data more than 1.5 × Interquartile Range (IQR) below Q1 and more than 1.5 × IQR above Q3 were classified as outliers and removed from analysis. According to the Shapiro Wilks normality test, data were not normally distributed. Permutation methods control false positives, allow the use of non-standard statistics and make only weak assumptions regarding the data [69]. Therefore, we decided to conduct a multiple linear regression using the permutation package Permuco in R [70]. STICSA state somatic anxiety was the dependent variable. Independent variables were STICSA trait cognitive, STICSA trait somatic, the STOMP music preferences factors (Reflective & Complex, Intense & Rebellious, Upbeat & Conventional, Energetic & Rhythmic) and the EPQR factors (Extraversion, Neuroticism, Psychoticism, Lie scale).

Participants were separated into moderate and high trait anxiety conditions according to previously established thresholds described in the Participants section [55]. Somatic and cognitive state anxiety reduction was calculated by subtracting the post-treatment STICSA state score from the pre-treatment STICSA state score for each participant. Pairwise Fisher Randomization Resampling tests (5000 iterations) also known as permutation tests were done comparing the treatment conditions for both somatic and cognitive state anxiety reduction. Permutation tests are a good way to control the type I error rate for multiple comparisons, it is non-parametric and so makes no assumptions about the underlying distribution of the data that are common in other inferential statistical tests [71–74]. For the PANAS, for each participant, the pre-treatment positive affect was subtracted from the post-treatment positive affect. The post-treatment negative affect was subtracted from the pre-treatment negative affect for each participant. Pairwise Fisher Randomization Resampling tests (FRT) (5000 iterations) also known as permutation tests were done comparing the treatment conditions for both positive and negative affect.

## Results

**Fig 2:**
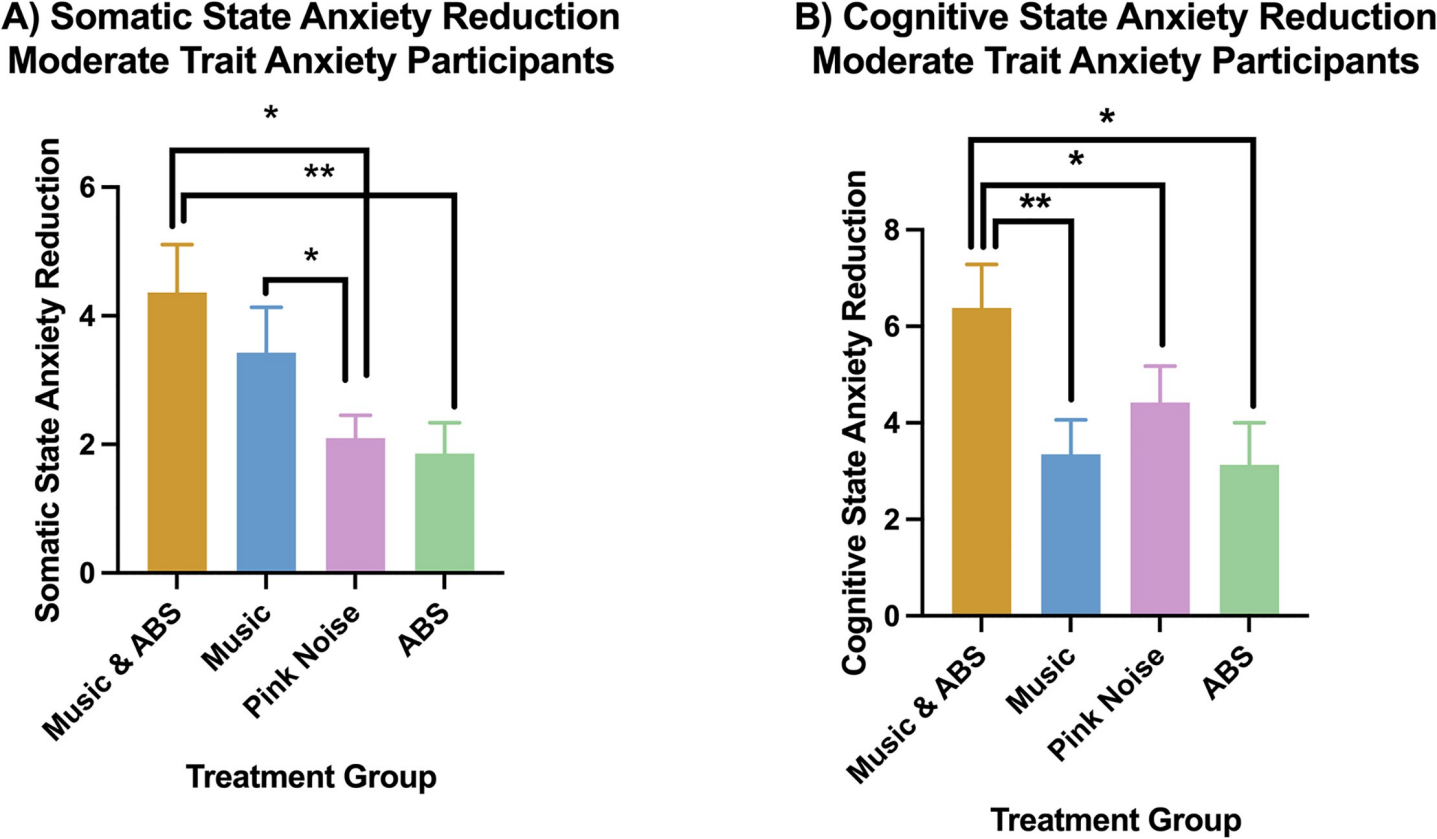
Mean somatic (A) and cognitive (B) state anxiety reduction in moderate trait anxiety participants. * Indicates p < 0.05 (FRT, 5000 iterations), ** indicates p < 0.01 (FRT, 5000 iterations). Error bars are standard deviations. Please see Table 2A and 2B for exact p values, effect sizes, power and degrees of freedom for each significant comparison.

In moderate trait anxiety participants, the combined and music-alone conditions had significantly higher somatic state anxiety reduction than the pink noise condition (Fig 2A). The combined condition also had a significantly higher somatic state anxiety reduction compared to the ABS condition (Fig 2A) and a significantly higher cognitive state anxiety reduction than the music-alone, ABS-alone and pink noise conditions (Fig 2B). We obtained the following mean anxiety reductions (± standard deviation). For somatic state anxiety (Fig 2A): Combined = 4.36 (3.49), Music = 3.43 (3.23), ABS = 1.86 (2.20), Pink Noise = 2.10 (1.64). For cognitive state anxiety (Fig 1B): Combined = 6.38 (4.14), Music = 3.35 (3.20), ABS = 3.13 (4.18), Pink Noise = 4.42 (3.83).

In high trait anxiety participants, the combined condition had a greater decrease in negative affect compared to the ABS-alone condition (p < 0.05). There were no other significant differences in any other pairwise comparisons for either positive or negative affect. We obtained the following mean changes in positive and negative affect (± standard deviation). For positive affect: Combined = -1.71 (8.74), Music = 2.29 (7.05), ABS = -1.39 (8.03), Pink Noise = -1.05 (8.66). For negative affect: Combined = -10.94 (8.12), Music = -7.57 (5.73), ABS = -5.22 (7.43), Pink Noise = -8.50 (10.55).

The multiple linear regression with state anxiety as the dependent variable and trait anxiety as an independent variable revealed a significant positive relationship between state anxiety reduction and trait anxiety of participants (p < 0.05) and a significant negative relationship between state anxiety reduction and a preference for Intense & Rebellious music preference factor (p < 0.05).

## Discussion

The combined and music-alone conditions had greater somatic state anxiety reduction than the pink-noise control condition in participants with moderate trait anxiety (Fig 2A). The effect size of these comparisons was large (Table 2A). One reason for these large effect sizes may be the use of slow tempo in music selections. Slow tempo music has been associated with reductions in respiration rate, heart rate, sweat production, body temperature, and muscle tension, the same physiological changes associated with reducing somatic anxiety [25,75–78]. Interestingly, we found no significant difference in somatic state anxiety reduction between the ABS and pink noise conditions in moderate trait anxiety participants (Fig 2A). This finding may be explained by the potential for pink noise to increase relaxation and sleep quality [64,79] coupled with the potential for theta ABS to increase negative affect [80,81].

**Table 2:** A: Additional statistical information for significant comparisons for somatic state anxiety reduction in moderate trait anxiety participants. B: Additional statistical information for significant comparisons for cognitive state anxiety reduction in moderate trait anxiety participants.

Comparison	Tail of Test	Exact p-value	Effect size (Cohen’s d)	95% CI for Effect Size	Power	Degrees of Freedom
Combined vs. ABS	Two-tailed	0.009	0.86	0.22–1.48	0.79	41
Combined vs. Pink Noise	One-tailed	0.04	0.83	0.20–1.45	0.84	41
Music vs. Pink Noise	One-tailed	0.05	0.52	-0.1–1.13	0.52	40
Comparison	Tail of Test	Exact p-value	Effect size (Cohen’s d)	95% CI for Effect Size	Power	Degrees of Freedom
Combined vs. Music	Two-tailed	0.01	0.82	0.17–1.45	0.72	39
Combined vs. ABS	Two-tailed	0.03	0.78	0.16–1.39	0.72	42
Combined vs. Pink Noise	One-tailed	0.05	0.49	-0.09–1.07	0.50	45

**Fig 3:**
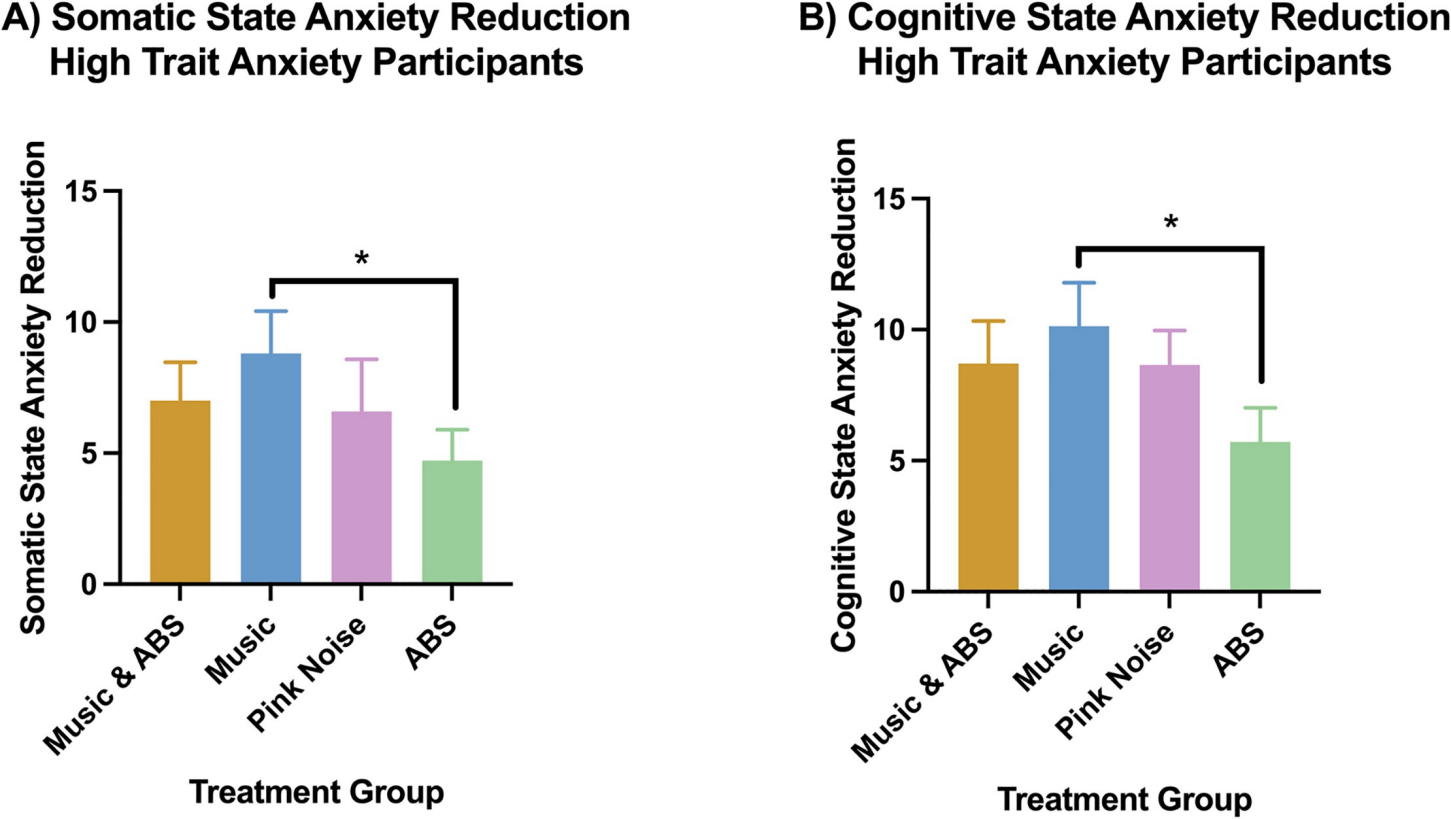
Mean somatic (A) and cognitive (B) state anxiety reduction in high trait anxiety participants. * Indicates p < 0.05 (FRT, 5000 iterations). Error bars are standard deviations. Please see Table 4A and 4B below for exact p values, tail of test, effect sizes, power and degrees of freedom for each significant comparison.

In high trait anxiety participants, the music-alone condition had significantly higher somatic (Fig 3A), and cognitive (Fig 3B) state anxiety reductions compared to the ABS-alone condition. There were no other significant differences between any of the other treatments. We obtained the following mean anxiety reductions (± standard deviation). For somatic state anxiety (Fig 3A): Combined = 7.00 (6.04), Music = 8.80 (6.25), ABS = 4.72 (4.96), Pink Noise = 6.60 (8.86). For cognitive state anxiety (Fig 3B): Combined = 8.71 (6.70), Music = 10.13 (6.42), ABS = 5.72 (5.52), Pink Noise = 8.65 (5.92).

**Fig 4:**
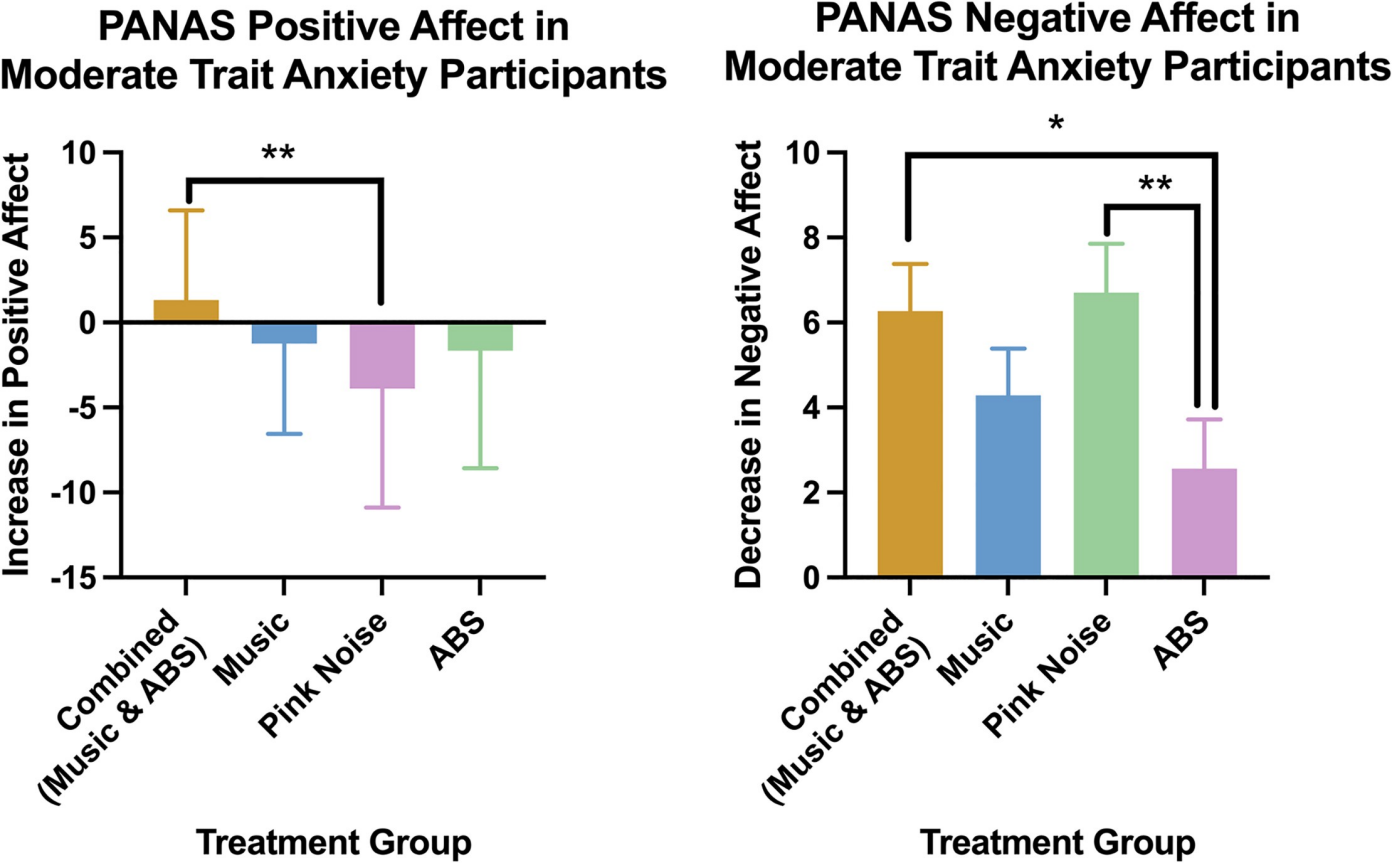
Mean positive affect increase (A) and negative affect decrease (B). ** indicates p < 0.01 (two tailed FRT, 5000 iterations). Error bars are standard deviations. Please see Table 3A and 3B in supplementary information for exact p values, effect sizes, power and degrees of freedom for each significant comparison.

In moderate anxiety participants, the combined condition also had significantly higher increase in positive affect compared to the pink noise condition (Fig 4A) and a significantly higher decrease in negative affect compared to the ABS-alone condition (Fig 4B). We obtained the following mean changes in positive and negative affect (± standard deviation). For positive affect (Fig 4A): Combined = 1.32 (5.28), Music = -1.24 (5.32), ABS = -1.65 (6.91), Pink Noise = 3.89 (6.99). For negative affect (Fig 3B): Combined = 6.27 (5.18), Music = 4.29 (5.03), ABS = 2.57 (5.55), Pink Noise = 6.70 (5.99).

In moderate trait anxiety participants, although the music condition did not lead to a significant reduction in cognitive anxiety compared to the pink noise control, the combined (music & ABS) condition did (Fig 2B). In addition, the combined condition compared to the pink noise control also had a medium effect size (Table 2B). We can speculate that the theta ABS component of the combined condition may have contributed to reductions in cognitive anxiety by entraining endogenous oscillations that are characteristic of relaxation [45,82]. Another possibility is that the ABS component increased interhemispheric coherence [83]. To investigate these possibilities, future studies should incorporate EEG in addition to self-report methods.

Prior studies demonstrated that ABS integrated with special carrier tones and algorithmic audio generation methods reduce general anxiety either with music or within the context of meditation [52–54]. However, these prior studies did not differentiate between somatic and cognitive anxiety, so it is difficult to determine the specific impact that the ABS may have had on cognitive anxiety.

In moderate trait anxiety participants, there was increased positive affect in the combined condition compared to the pink-noise condition (Fig 4A). This comparison had a medium effect size (Table 3A). Music listening increases positive affect which may help explain this result [84,85]. There was a larger decrease in negative affect in the combined and pink noise conditions compared to the ABS-alone condition (Fig 4B). Both comparisons had a medium effect size (Table 3B). Theta ABS increases the Profile of Mood States depression subscale compared to pink noise and increases negative affect compared to beta ABS [80,81]. These prior studies did not specifically examine a moderate or high trait anxiety population and since trait anxiety may affect physiological responses to music [50,51], this could be one reason for the mixed results across our outcome measures.

**Table 3:** A: Additional statistical information for significant comparisons for positive affect increase in moderate trait anxiety participants. B: Additional statistical information for significant comparisons for negative affect decrease in moderate trait anxiety participants.

Comparison	Tail of Test	Exact p-value	Effect size (Cohen’s d)	95% CI for Effect Size	Power	Degrees of Freedom
Combined vs. Pink Noise	Two-tailed	0.005	0.48	0.23–1.42	0.35	43
Comparison	Tail of Test	Exact p-value	Effect size (Cohen’s d)	95% CI for Effect Size	Power	Degrees of Freedom
Combined vs. ABS	Two-tailed	0.016	0.69	0.08–1.29	0.62	43
Pink Noise vs. ABS	Two-tailed	0.01	0.72	0.14–1.28	0.70	48

In high trait anxiety participants, the music condition had a larger somatic and cognitive state anxiety reduction compared to the ABS condition (Fig 3A and 3B). These comparisons had a medium effect size (Table 4A and 4B). But there were no significant differences in somatic or cognitive anxiety reduction between combined, music-alone and pink noise conditions. Pink noise deepens and improves sleep quality [64,79]. This combined with the fact that theta ABS has been shown to in some cases to increase negative affect [80,81], may be the reason that the music with ABS condition and music conditions did not have larger anxiety reductions compared to the pink noise condition. For high trait anxiety participants, transitioning from beta to alpha ABS within the first 5 minutes of the treatment may not cause the increase in negative affect seen with theta ABS, but does not appear to reduce anxiety compared to music alone [53]. High trait anxiety participants may require longer and multiple sessions to achieve a reduction in anxiety.

**Table 4:** A: Additional statistical information for significant comparisons for somatic state anxiety reduction in high trait anxiety participants. B: Additional statistical information for significant comparisons for cognitive state anxiety reduction in high trait anxiety participants.

Comparison	Tail of Test	Exact p-value	Effect size (Cohen’s d)	95% CI for Effect Size	Power	Degrees of Freedom
Music vs. ABS	Two-tailed	0.04	0.72	0.02–1.43	0.52	31
Comparison	Tail of Test	Exact p-value	Effect size (Cohen’s d)	95% CI for Effect Size	Power	Degrees of Freedom
Music vs. ABS	Two-tailed	0.04	0.74	0.03–1.45	0.53	31

In high trait anxiety participants, the combined condition had a significantly higher reduction in negative affect compared to the ABS condition. Music decreases negative affect [84,85], whereas theta ABS alone may increase negative affect [80,81]. Therefore, combining music with ABS may cause a decrease in negative affect when compared to the ABS-alone condition. Some individuals may also find the persistent hum of the ABS-alone condition irritating compared to the combined condition where this ABS hum is masked with the music. The pink noise condition also had a significantly higher reduction in negative affect compared to the ABS condition. This may be explained by the fact that pink noise increases sleep quality and relaxation [64,79] and participants may have found the persistent hum of the ABS only condition irritating.

Although much of the research involving ABS points to anxiety reduction, increased relaxation and improved cognition and attention, the effectiveness of ABS has not been supported in all studies [86–90]. This inconsistency in experimental results is likely due to several factors which include frequency used, type of sound used to mask the binaural beat, trait anxiety, personality factors such as extraversion and duration of exposure [49–51,91–93]. In this study we accounted for these factors statistically and through experimental design which should help increase the reproducibility of this study. Specifically, we took into account personality traits and trait anxiety of our participants. Personality traits such as extraversion are related to mesostriatal dopamine levels which have been found to determine the effectiveness of gamma ABS in improving cognition [49]. In this study, we used theta ABS and found no significant effects of extraversion on the anxiety reduction effectiveness of our music with ABS or ABS conditions, but we did see a significant effect of trait anxiety on anxiety reduction effectiveness.

Participants in all conditions experienced pre-post reductions in somatic and cognitive state anxiety suggesting the presence of demand/expectancy characteristics. However, given that some conditions (Combined and music) resulted in significantly greater anxiety reduction than others (pink noise control), this suggests that experimental condition plays a significant role over the presence of demand characteristics. Nevertheless, in future studies the Credibility and Expectancy Questionnaire [94] may be administered to further examine the role of demand characteristics. Comorbidities such as depression often occur in people with anxiety symptoms. However, time constraints and limitations of the Prolific platform with respect to recruiting enough participants with and without anxiety comorbidities such as depression precluded extensive data collection beyond the primary domain of interest (anxiety). Although, comorbidities such as depression can affect anxiety treatment outcomes, particularly in older populations [95]. In some cases, particularly in younger populations comorbidities do not seem to affect treatment outcomes [96,97]. This is one of the first few studies examining the anxiety reducing potential of music and ABS. Based on prior work we wanted to control for potential confounding effects of personality, music preferences and musical experience [49–51]. These additional questionnaires took up considerable amounts of the participants’ time, so a limitation in this study is that there was not any time left to administer additional anxiety measures without compromising data quality, particularly given the online nature of data collections. In addition, because the study was conducted online, we could not do a hetero-applied medical evaluation assessment and there is also inherently a lack of control over certain variables such as timing of when participants were taking their anxiolytics. The sample population of this study were on anxiolytics with the majority being on SSRIs (Tables A & B in S1 File). SSRIs may inhibit emotional responses [98] which in turn may impact the responsiveness to music/sound based treatments. This limits the generalizability of our findings to those taking anxiolytics. However, we hope to address this in a future study examining the anxiety-reducing potential of sound-based treatments on both anxiolytic and non-medicated populations.

## Conclusion

In moderate trait anxiety participants, combined and music-alone conditions led to significant reductions in somatic anxiety compared to ABS-alone and pink noise conditions. Similarly, the combined condition significantly reduced cognitive anxiety compared to the ABS-alone and pink noise conditions. In high trait anxiety participants, the music treatment significantly reduced somatic and cognitive anxiety compared to the ABS treatment, however, the pattern of results was difficult to interpret because of the lack of differences with the pink noise condition. We hypothesize that high trait anxiety participants may require longer and more frequent music treatments over time in order to achieve the same reductions in anxiety as moderate trait anxiety participants. We hope to examine this hypothesis in a future longitudinal study. The implications of this work are immense as many people who suffer from anxiety do not respond to standard treatments and fail to seek treatment and this simple and easily distributable method of potentially reducing anxiety may help serve this segment of the population.

## Supporting information

S1 Checklist CONSORT 2010 checklist. (DOCX)

S1 File Medication information for moderate and high trait anxiety participants. (DOCX)

S2 File (PDF)

S3 File (PDF)

S4 File (PDF)

S5 File (PDF)
