# Supplementary material for: The effects of music & auditory beat stimulation on anxiety: A randomized clinical trial
Source: PLoS One. 2022 Mar 9;17(3):e0259312. doi: 10.1371/journal.pone.0259312 (PMC8906590; doi:10.1371/journal.pone.0259312)
Supplement: S1 File — (DOCX) [file pone.0259312.s002.docx]

**S1 Medication Information. Medication Information for Moderate and High Trait Anxiety Participants**

Table A: Medication Information for Moderate Trait Anxiety Participants

| Medication Class | Not Disclosed | SSRI | SNRI | BB | BZ | AH | AP | NDRI | AC | NaSSA | 5-HT-RA | TA | ST | Other |
| --- | --- | --- | --- | --- | --- | --- | --- | --- | --- | --- | --- | --- | --- | --- |
| # of Participants | 32 | 39 | 2 | 5 | 6 | 1 | 1 | 2 | 0 | 1 | 0 | 1 | 1 | 1 |

Table B: Medication Information for High Trait Anxiety Participants

| Medication Class | Not Disclosed | SSRI | SNRI | BB | BZ | AH | AP | NDRI | AC | NaSSA | 5-HT-RA | TA | Other |
| --- | --- | --- | --- | --- | --- | --- | --- | --- | --- | --- | --- | --- | --- |
| # of Participants | 20 | 27 | 2 | 10 | 5 | 1 | 3 | 1 | 2 | 1 | 1 | 1 | 1 |

Abbreviations: SSRI = Selective Serotonin Reuptake Inhibitor, SNRI = Serotonin Norepinephrine Reuptake Inhibitor, BB = Beta Blocker, BZ = Benzodiazepine, AH = Antihistamine, AP = Antipsychotic, NDRI = Norepinephrine-Dopamine Reuptake Inhibitor, AC = Anticonvulsant, NaSSA = Noradrenergic and specific serotonergic antidepressant, 5-HT-RA = Serotonin 5-HT1A Receptor Agonist, ST = Stimulant, TA = Tricyclic Antidepressants, Other = general mention of antidepressants
