# Supplementary material for: The effects of music & auditory beat stimulation on anxiety: A randomized clinical trial
Source: PLoS One. 2022 Mar 9;17(3):e0259312. doi: 10.1371/journal.pone.0259312 (PMC8906590; doi:10.1371/journal.pone.0259312)
Supplement: S2 File — (PDF) [file pone.0259312.s003.pdf]

ClinicalTrials.gov PRS
Protocol Registration and Results System

ClinicalTrials.gov PRS DRAFT Receipt (Working Version)
Last Update: 12/08/2021 13:45

ClinicalTrials.gov ID: [Not yet assigned]

Study Identification

Unique Protocol ID: REB 2020-068

Brief Title: The Effects of Music & Auditory Beat Stimulation on Anxiety

Official Title: The Effects of Music & Auditory Beat Stimulation on Anxiety

Secondary IDs:

Study Status

Record Verification: November 2021

Overall Status: Completed

Study Start: July 9, 2020 [Actual]

Primary Completion: February 2, 2021 [Actual]

Study Completion: February 2, 2021 [Actual]

Sponsor/Collaborators

Sponsor: Ryerson University

Responsible Party: Principal Investigator
Investigator: Frank Russo [frusso]
Official Title: Professor
Affiliation: Ryerson University

Collaborators: Lucid, Inc.
Mitacs

Oversight

U.S. FDA-regulated Drug: No

U.S. FDA-regulated Device: No

U.S. FDA IND/IDE: No

Human Subjects Review: Board Status: Approved
Approval Number: REB 2020-068
Board Name: Ryerson University Research Ethics Board
Board Affiliation: Ryerson University
Phone: 4169795000
Email: rebchair@ryerson.ca
Address:

Office of the Vice-President, Research & Innovation

1 Dundas Street West, 11th Floor
Toronto, Ontario M5G 1Z3

Data Monitoring: No

FDA Regulated Intervention: No

Study Description

Brief Summary: Anxiety is a growing problem and has been steadily increasing, particularly in the adolescent and young adult populations in the past 24 years. Music and auditory beat stimulation (ABS) in the theta frequency range (4-7 Hz) are sound-based anxiety treatments that have been independently investigated in prior studies. Here, the anxiety-reducing potential of calm music combined with theta ABS was examined in a large sample of participants. Participants taking anxiolytics (n = 163) were randomly assigned to a single 24-minute session of sound-based treatment: combined (music & ABS), music-alone, ABS-alone, or pink noise (control). Pre- and post-intervention somatic and cognitive state anxiety measures (STICSA State) were collected along with trait anxiety (STICSA Trait), personality measures (Short Form Eysenck Personality Inventory) and musical preferences (Short Test of Music Preferences).

Detailed Description: In this study, the investigators examined and compared the effectiveness of ABS in the theta range, calm music playlist curated by an affective music recommendation system, and the combination of ABS and the same music to reduce anxiety and stress levels (as measured by the State Trait Inventory for Cognitive and Somatic Anxiety (STICSA)) compared to a control condition (pink noise). Prior work has demonstrated that ABS and music both reduce anxiety when presented on their own. It is hypothesized that music with ABS will lead to significantly lower anxiety levels and increased calmness compared to the other experimental conditions. Approximately 163 participants were recruited from the Prolific online participant pool (https://www.prolific.co). The experiment was conducted on the Qualtrics survey platform, and the experimental treatment was provided with the LUCID Research App. After reading and agreeing with the consent form, participants provided their Prolific ID and then filled out the Short Test of Music Preferences (STOMP), Queen’s Music Questionnaire, Anxiety coping method’s questionnaire, were asked to list any medications they are currently taking (including cannabis) and completed the Positive and Negative Affect Scale (PANAS), Self-Assessment Manikin (SAM), Eysenck Personality Questionnaire and the State Trait Inventory of Cognitive and Somatic Anxiety (STICSA) trait and state questionnaires. Participants were then randomly assigned to one of four treatment groups where they listened to either music, music and auditory beat stimulation (ABS), auditory beat stimulation (ABS) alone, or pink noise for 24 minutes. Participants then received instructions on how to download the LUCID Research app on their iOS device or access the LUCID Research App through a virtual machine using their computer. Participants listened to their randomly assigned treatment for 24 minutes. Participants then completed their post-intervention questionnaires which included: the STICSA state version, SAM and PANAS. The investigators’ hypotheses were that the combined, music alone and ABS alone conditions would experience a greater reduction in somatic and cognitive state anxiety compared to the pink noise control condition. These hypotheses were pre-registered using the Open Science Framework (Registration DOI: https://doi.org/10.17605/OSF.IO/VHCA5) and were based upon previous studies showing that ABS and music listening are capable of reducing anxiety. The investigators had no specific predictions for moderate and high trait anxiety participants, but their pre-registration noted their intention to recruit from both of these populations.

Conditions

Conditions: Anxiety

Keywords: Anxiety
Auditory Beat Stimulation
Binaural Beats
Mental Health
Music
Neuroscience
Psychology

Study Design

Study Type: Interventional

Primary Purpose: Treatment

Study Phase: N/A

Interventional Study Model: Parallel Assignment
Parallel Assignment Participants taking anxiolytics (n = 163) were randomly assigned to a single 24- minute session of sound-based treatment: combined (Music with theta auditory beat stimulation), music-alone, theta auditory beat stimulation-alone, or pink noise (control). Pre- and post-intervention somatic and cognitive state anxiety measures (STICSA State) were collected along with trait anxiety (STICSA Trait), personality measures (Short Form Eysenck Personality Inventory) and musical preferences (Short Test of Music Preferences).

Number of Arms: 4

Masking: None (Open Label)

Allocation: Randomized

Enrollment: 163 [Actual]

Arms and Interventions

Arms	Assigned Interventions
Experimental: Music & Auditory Beat Stimulation<br>Participants listened to calm music with theta auditory beat stimulation for 24 minutes	Behavioral: Music & Auditory Beat Stimulation<br>Listening to calm music and auditory beat stimulation Participants listened to calm music with theta auditory beat stimulation for 24 minutes
Active Comparator: Music Alone<br>Participants listened to calm music for 24 minutes	Behavioral: Music Alone<br>Listening to calm music Participants listened to calm music for 24 minutes
Active Comparator: Auditory Beat Stimulation<br>Participants listened to theta auditory beat stimulation for 24 minutes	Behavioral: Auditory Beat Stimulation<br>Listening to theta auditory beat stimulation Participants listened to theta auditory beat stimulation for 24 minutes
Sham Comparator: Pink Noise<br>Participants listened to pink noise for 24 minutes	Behavioral: Pink Noise<br>Listening to pink noise Participants listened to pink noise for 24 minutes

Outcome Measures

Primary Outcome Measure:

1. Anxiety: State Trait Inventory for Cognitive and Somatic Anxiety (STICSA)
   The State Trait Anxiety Inventory for Cognitive and Somatic Anxiety has good reliability and validity as a measure of state and trait cognitive and somatic anxiety. The minimum score is 10 and the maximum is 40. Higher scores indicate higher anxiety (worse outcome). But in this study the post-intervention anxiety score is subtracted from the pre-intervention anxiety score, giving a measure of anxiety reduction. In the case of this anxiety reduction measure, higher anxiety reduction scores would indicate a better outcome.

   [Time Frame: 24 minutes]

Secondary Outcome Measure:

2. Mood: Positive and Negative Affect Scale (PANAS)
   The Positive and Negative Affect Scale has good reliability and validity and has been widely used in many studies to assess mood. This scale generates two scores: 1) Positive affect (higher score indicates a better outcome), scores range from 10-50. 2) Negative affect (higher score indicates worse outcome), scores range from 10-50.

   [Time Frame: 24 minutes]

Eligibility

Minimum Age: 18 Years

Maximum Age:

Sex: All

Gender Based: No

Accepts Healthy Volunteers: No

Criteria: Inclusion Criteria:

- Adults (18+)
- Must be taking anxiety medication
- Self-identified normal hearing
- No known cardiac issues
- No known epilepsy/seizures

  - Have access to an iOS device (iPhone or iPad) to run the Research Application

Exclusion Criteria:

- Adults younger than 18
- Not taking anxiety medication
- Have known cardiac issues

  - Do not have access to an iOS device (iPhone or iPad) to run the Research Application
- Have known epilepsy/seizures

Contacts/Locations

Central Contact Person: Adiel Mallik, PhD
Telephone: 4169795000 Ext. 554989
Email: adiel.mallik@ryerson.ca

Central Contact Backup:

Study Officials: Frank A Russo, PhD
Study Director
Ryerson University

Locations: **Canada, Ontario**
Ryerson University
Toronto, Ontario, Canada, M5B 2K3
Contact: Karla Kovacek, BA 4169795000 Ext. 554989
karla.kovcek@ryerson.ca
Contact: Kay Wright-Whyte, MSc 41697950004989 Ext. 554989
kww@ryerson.ca

IPDSharing

Plan to Share IPD: Yes
We have registered the project on Open Science Framework and will make all elements of IPD open to the public. Please see: https://osf.io/efya2/ Study protocol and analysis plan: https://doi.org/10.17605/ OSF.IO/VHCA5 Consent form: https://osf.io/efya2/

Supporting Information:
Study Protocol
Statistical Analysis Plan (SAP)
Informed Consent Form (ICF)

Time Frame:
Data are available now and will be for 7 years post data collection completion as per our REB approval: approximately January 2028

Access Criteria:
There is no specific access criteria for the IPD on OSF. It is freely and openly available to anyone.

URL: https://osf.io/efya2/

References

Citations:
Bados A, Gómez-Benito J, Balaguer G. The state-trait anxiety inventory, trait version: does it really measure anxiety? J Pers Assess. 2010 Nov;92(6):560-7. doi: 10.1080/00223891.2010.513295. PubMed 20954057

Gray EK, Watson, D. Assessing positive and negative affect via self-report. In: Coan JA, Allen, J.J.B., editor. Handbook of emotion elicitation and assessment. New York, NY: Oxford University Press; 2007.

Grös DF, Antony MM, Simms LJ, McCabe RE. Psychometric properties of the State-Trait Inventory for Cognitive and Somatic Anxiety (STICSA): comparison to the State-Trait Anxiety Inventory (STAI). Psychol Assess. 2007 Dec;19(4):369-81. PubMed 18085930

Phillips SP, Yu J. Is anxiety/depression increasing among 5-25 year-olds? A cross-sectional prevalence study in Ontario, Canada, 1997-2017. J Affect Disord. 2021 Mar 1;282:141-146. doi: 10.1016/j.jad.2020.12.178. Epub 2020 Dec 30. PubMed 33418360

Watson D, Clark LA, Tellegen A. Development and validation of brief measures of positive and negative affect: the PANAS scales. J Pers Soc Psychol. 1988 Jun;54(6):1063-70. PubMed 3397865

Davis WB, Thaut MH. The Influence of Preferred Relaxing Music on Measures of State Anxiety, Relaxation, and Physiological Responses. Journal of Music Therapy. 1989;26(4):168-87. doi: 10.1093/jmt/26.4.168.

Isik BK, Esen A, Büyükerkmen B, Kilinç A, Menziletoglu D. Effectiveness of binaural beats in reducing preoperative dental anxiety. Br J Oral Maxillofac Surg. 2017 Jul;55(6):571-574. doi: 10.1016/j.bjoms.2017.02.014. Epub 2017 Mar 18. PubMed 28325532

McConnell PA, Froeliger B, Garland EL, Ives JC, Sforzo GA. Auditory driving of the autonomic nervous system: Listening to theta-frequency binaural beats post-exercise increases parasympathetic activation and sympathetic withdrawal. Front Psychol. 2014 Nov 14;5:1248. doi: 10.3389/fpsyg.2014.01248. eCollection 2014. PubMed 25452734

Padmanabhan R, Hildreth AJ, Laws D. A prospective, randomised, controlled study examining binaural beat audio and pre-operative anxiety in patients undergoing general anaesthesia for day case surgery. Anaesthesia. 2005 Sep;60(9):874-7. PubMed 16115248

Wahbeh H, Calabrese C, Zwickey H. Binaural beat technology in humans: a pilot study to assess psychologic and physiologic effects. J Altern Complement Med. 2007 Jan-Feb;13(1):25-32. PubMed 17309374

Yusim A, Grigaitis J. Efficacy of Binaural Beat Meditation Technology for Treating Anxiety Symptoms: A Pilot Study. J Nerv Ment Dis. 2020 Feb;208(2):155-160. doi: 10.1097/NMD.0000000000001070. PubMed 31977827

Bringman H, Giesecke K, Thörne A, Bringman S. Relaxing music as pre-medication before surgery: a randomised controlled trial. Acta Anaesthesiol Scand. 2009 Jul;53(6):759-64. doi: 10.1111/j.1399-6576.2009.01969.x. Epub 2009 Apr 14. PubMed 19388893

Links:

Available IPD/Information: Type: Individual Participant Data Set
URL: https://osf.io/efya2/

Type: Study Protocol
URL: https://doi.org/10.17605/OSF.IO/VHCA5

Type: Statistical Analysis Plan
URL: https://doi.org/10.17605/OSF.IO/VHCA5

Type: Informed Consent Form
URL: https://osf.io/efya2/

Documents

Study Protocol
Document Date: March 18, 2021
Uploaded: 11/26/2021 16:54

Statistical Analysis Plan
Document Date: March 18, 2021
Uploaded: 11/26/2021 16:57

Informed Consent Form
Document Date: March 18, 2021
Uploaded: 11/26/2021 16:58
