# Supplementary material for: The effects of music & auditory beat stimulation on anxiety: A randomized clinical trial
Source: PLoS One. 2022 Mar 9;17(3):e0259312. doi: 10.1371/journal.pone.0259312 (PMC8906590; doi:10.1371/journal.pone.0259312)
Supplement: S4 File — (PDF) [file pone.0259312.s005.pdf]

The Effects of Music and Auditory Beat Stimulation on Anxiety

Statistical Analysis Plan

NCT#:

Date: 2021-11-26

Statistical Analysis Plan

For each participant the post-experimental treatment STICSA, PANAS and SAM Valence and arousal scores will be subtracted from the pre-experimental treatment scores. Shapiro-Wilks normality and homogeneity of variances tests will be done on the STICSA, PANAS and SAM datasets, and homogeneity of covariances tests will be done on music preferences, and personality type datasets to determine if assumptions of the Multivariate Analysis of Covariance (MANCOVA) are satisfied. If these assumptions are satisfied, a MANCOVA over the factor of experimental condition will be performed with music preferences, spontaneous blink rate and personality type as covariates and the relative differences (Pre-treatment – Post-treatment) of STICSA, PANAS and SAM Valence and arousal scores as dependent variables. Post-hoc Tukey's HSD multiple comparisons tests will be done comparing the LUCID music with theta ABS condition with all of the other experimental conditions. Further post-hoc Tukey's HSD multiple comparisons tests will be done comparing the theta ABS and LUCID music conditions with the pink noise condition. Statistically accounting for music preferences, spontaneous blink rate, personality type, age and gender as covariates should reduce some variability in the analysis.

If the data do not satisfy the assumptions for the MANCOVA, permutations tests, specifically the lmPerm package in R will be used to conduct a permutation version of the repeated measures ANCOVA for each dependent variable (STICSA Somatic, STICSA Cognitive, Positive Affect (PANAS), Negative Affect (PANAS) and SAM Valence and arousal scores), with time (pre and post intervention) as the within subjects variable and experimental condition as the between subjects factor. Post-hoc permutation tests will be done comparing the LUCID music with theta binaural beats condition with all of the other experimental conditions for each dependent variable. Permutation tests are a good way to control the type I error rate for multiple comparisons, it is non-parametric and so makes no assumptions about the underlying distribution of the data that are common in other inferential statistical tests (Camargo, Azuaje, Wang, & Zheng, 2008; Good, 1994; Kuehl, 2000).

References

Camargo, A., Azuaje, F., Wang, H., & Zheng, H. (2008). Permutation – based statistical tests for multiple hypotheses. *Source Code for Biology and Medicine*, *3*(1), 15. doi:10.1186/1751-0473-3-15

Good, P. (1994). *Permutation tests: A practical guide to resampling methods for testing hypotheses*. New York: Springer Science + Business Media.

Kuehl, R. O. (2000). *Design of experiments: Statistical Principles of Research Design* (2nd ed.). Pacific Grove, California: Duxbury Press.
