# Supplementary material for: The effects of music & auditory beat stimulation on anxiety: A randomized clinical trial
Source: PLoS One. 2022 Mar 9;17(3):e0259312. doi: 10.1371/journal.pone.0259312 (PMC8906590; doi:10.1371/journal.pone.0259312)
Supplement: S5 File — (PDF) [file pone.0259312.s006.pdf]

The Effects of Music and Auditory Beat Stimulation on Anxiety
Study Protocol

Approved by the Ryerson Ethics Board: REB 2020-068 on March 26th, 2020, renewal approval: March 18th, 2021

NCT#:

Date: 2021-11-26

Study Protocol

Participants

Participants (n=318) who are taking anti-anxiety medication will be recruited from Prolific, an online research recruitment platform. A power analysis was conducted drawing effect sizes from a previous study that examined how anxiety levels (measured by the STAI), were affected under the following experimental conditions: binaural beats, music with binaural beats and no-intervention (Padmanabhan et al., 2005). The power analysis indicated that to achieve a power of 0.80 at a significance of p = 0.05, a minimum of 45 participants in each treatment group are required (Padmanabhan et al., 2005). An additional 10 participants will be recruited to account for potential participant attrition. Participants will be pre-screened for moderate to severe anxiety using the STICSA trait questionnaire (Grös, Antony, Simms, & McCabe, 2007). Participants will then be randomly assigned to one treatment arm.

Experimental treatment conditions

Each of the experimental treatment conditions below will be 24 minutes in duration, as this seems to be the exposure time needed for binaural beats to reduce anxiety levels and for general effectiveness of binaural beats in other areas such as cognition (Garcia-Argibay, 2019; Isik et al., 2017).

1. Pink noise (control condition)
2. Theta ABS
3. Theta ABS with music generated by LUCID’s calm mode
4. Music generated by LUCID’s calm mode

Procedure

After reading and signing the consent form and going over experimental expectations, participants will be asked to fill out the Short Test of Music Preferences (STOMP), Queen’s Music Questionnaire, the Positive and Negative Affect Scale (PANAS), Self-Assessment Manikin (SAM), Eysenck Personality Questionnaire and the STICSA state version. Participants will then undergo their respective listening condition. After undergoing experimental treatment, participants will complete the following questionnaires: STICSA state version, SAM and the PANAS.

Outcome measures and questionnaires

Participants show a more pleasurable emotional response to their own preferred music compared to experimenter-selected music (Davis & Thaut, 1989; Thaut & Davis, 1993). We therefore decided to incorporate the Short Test of Music Preferences (STOMP) as a covariate in our analyses (Rentfrow & Gosling, 2003). The Queen’s Music Questionnaire will be administered to assess musical education and daily music exposure of each participant, which will be used as a covariate in our analyses. The Eysenck Personality Questionnaire will be used to determine participants’ extraversion and introversion (Rocklin & Revelle, 1981). The State Trait Inventory for Cognitive and Somatic Anxiety (STICSA) will be used to pre-screen high trait anxiety participants (trait version) for participation in this study (Grös et al., 2007).

The state version of STICSA will be used to assess state anxiety before and after experimental treatment. This will serve as the primary outcome measure. The Positive and Negative Affect Scale (PANAS) will be used to assess mood (Watson, Clark, & Tellegen, 1988) before and after experimental treatment. The Self-Assessment Manikin (SAM)

questionnaire will be used to assess valence and arousal of the participant before and after experimental treatment (Bradley & Lang, 1994).

References

Bradley, M. M., & Lang, P. J. (1994). Measuring emotion: The self-assessment manikin and the semantic differential. Journal of Behavior Therapy and Experimental Psychiatry, 25(1), 49-59. doi:https://doi.org/10.1016/0005-7916(94)90063-9

Bringman, H., Giesecke, K., Thörne, A., & Bringman, S. (2009). Relaxing music as pre-medication before surgery: A randomised controlled trial. Acta Anaesthesiologica Scandinavica, 53(6), 759-764.

Davis, W. B., & Thaut, M. H. (1989). The Influence of Preferred Relaxing Music on Measures of State Anxiety, Relaxation, and Physiological Responses. Journal of Music Therapy, 26(4), 168-187. doi:10.1093/jmt/26.4.168

Garcia-Argibay, M. (2019). Efficacy of binaural auditory beats in cognition, anxiety, and pain perception: a meta-analysis. Psychological Research, 83(2), 357-372. doi:10.1007/s00426-018-1066-8

Grös, D. F., Antony, M. M., Simms, L. J., & McCabe, R. E. (2007). Psychometric properties of the State-Trait Inventory for Cognitive and Somatic Anxiety (STICSA): Comparison to the State-Trait Anxiety Inventory (STAI). Psychological assessment, 19(4), 369.

Isik, B., Esen, A., Büyükerkmen, B., Kilinç, A., & Menziletoglu, D. (2017). Effectiveness of binaural beats in reducing preoperative dental anxiety. British Journal of Oral and Maxillofacial Surgery, 55(6), 571-574.

Padmanabhan, R., Hildreth, A. J., & Laws, D. (2005). A prospective, randomised, controlled study examining binaural beat audio and pre-operative anxiety in patients undergoing general anaesthesia for day case surgery*. Anaesthesia, 60(9), 874-877. doi:10.1111/j.1365-2044.2005.04287.x

Rentfrow, P. J., & Gosling, S. D. (2003). The do re mi's of everyday life: The structure and personality correlates of music preferences. Journal of Personality and Social Psychology, 84(6), 1236.

Rocklin, T., & Revelle, W. (1981). The measurement of extroversion: A comparison of the Eysenck Personality Inventory and the Eysenck Personality Questionnaire. British Journal of Social Psychology, 20(4), 279-284. doi:10.1111/j.2044-8309.1981.tb00498.x

Thaut, M. H., & Davis, W. B. (1993). The influence of subject-selected versus experimenter-chosen music on affect, anxiety, and relaxation. Journal of Music Therapy, 30(4), 210-223.

Wahbeh, H., Calabrese, C., Zwickey, H., & Zajdel, D. (2007). Binaural beat technology in humans: a pilot study to assess neuropsychologic, physiologic, and electroencephalographic effects. *The Journal of Alternative and Complementary Medicine*, *13*(2), 199-206.

Watson, D., Clark, L. A., & Tellegen, A. (1988). Development and validation of brief measures of positive and negative affect: The PANAS scales. Journal of Personality and Social Psychology, 54(6), 1063-1070.
